# Prefectural Adequacy of Opioid Availability for Cancer Pain and Its Determinants in Japan: A Preliminary Study

**DOI:** 10.31662/jmaj.2020-0037

**Published:** 2020-10-02

**Authors:** Kenji Azuma, Hiroaki Abe, Jun Hozumi, Reo Inoue, Mitsuru Konishi, Rikuhei Tsuchida, Masae Ando, Kosuke Saita, Masahiko Sumitani

**Affiliations:** 1 Department of Pain and Palliative Medicine, The University of Tokyo Hospital, Tokyo, Japan; 2 Department of Anesthesiology and Pain Relief Center, The University of Tokyo Hospital, Tokyo, Japan

**Keywords:** Cancer pain, opioid analgesics, adequate consumption, palliative care, primary care

## Abstract

**Introduction:**

Opioid analgesics are the mainstay of cancer pain management. The annual opioid consumption globally indicates adequate opioid availability and the quality of palliative care. We investigated the current situation regarding the adequacy of opioid availability in individual prefectures in Japan and explored the determinants of adequacy.

**Methods:**

We analyzed nationwide databases open to public inspection depicting the current Japanese healthcare situation. Opioid consumption for cancer pain was estimated from oxycodone and morphine data in the nationwide database. On the basis of the World Health Organization recommendations, we calculated adequacy based on the annual cancer deaths in each prefecture in 2013 and 2015. We investigated the associations between adequacy and either outpatient medical expenditure for hypertension and diabetes as a proxy of primary care practice or ratios of these risk holders in community. Outpatient medical expenditures for musculoskeletal disorders and neoplasms were also investigated.

**Results:**

The nationwide adequacy of opioid availability was approximately 75%. The largest gaps in adequacy between prefectures were more than 65%. The adequacy correlated with expenditure but not local volumes of hypertension and diabetes in both years. The other two expenditures did not relate to opioid availability.

**Conclusions:**

Although precise data are required, our preliminary findings indicate that primary care practice is the key regulator of adequate opioid availability. Opioid adequacy in Japan is thus delayed in matching the global standard, and gaps in opioid adequacy among prefectures should be bridged rapidly to expand universal access to effective palliative care and cancer pain relief.

## Introduction

Pain is one of the most frequent symptoms among patients with terminal cancer. The prevalence rate of cancer pain is 64% in metastatic, advanced, or terminal disease. Furthermore, pain has been reported in 33% of patients just after curative treatment and 59% of patients during anticancer treatment ^(1)^. Pain-associated distress experienced by those with cancer pain can cause anxiety, depression, and changes in social function. Indeed, not only physical function but also social function and roles have been found to be lower in patients with cancer pain than in the general population. As a result, the overall health-related quality of life (QOL) of cancer pain patients is severely impaired ^(2)^. According to the original cancer pain relief guidelines for improving cancer patients’ QOL proposed by the World Health Organization (WHO) in 1986 ^(3)^, opioid analgesics have been the mainstay of analgesic therapy. The indications for strong opioids have recently been expanded to mild-moderate cancer pain, in addition to conventional moderate-severe cancer pain, because of their strong analgesic potency and comparatively mild adverse effects ^(4)^. However, in many nations, there are still several barriers to opioid availability, including public health policies, laws, and education of healthcare professionals ^(5)^. Most nations with significant barriers to opioid availability are not developed countries in which public health policies, laws, and education of healthcare professionals are generally mature ^(6)^. Such national maturity has been indexed and reported by the United Nations ^(7)^. In fact, opioid consumption was directly associated with indices of individual and social wellness ^(8), (9)^. Considering this, the total opioid consumption per capita at regional, national, and global levels has been proposed and widely accepted as a proxy marker of adequate opioid availability and the quality of palliative care worldwide ^(10)^. On the basis of the opioid consumption per capita, (1) the formulation and implementation of legal and regulatory guidelines, covering all service providers who participate in palliative care and pain treatment; (2) public education and awareness-building campaigns of opioids as essential drugs for cancer pain treatment; and (3) development and implementation of comprehensive palliative care and pain treatment guidelines, as well as national plans, have been used to expand universal access to effective palliative care and cancer pain relief. For instance, such national commitment to improving opioid availability in Serbia successfully increased opioid consumption by more than 30% between 2006 and 2010 ^(9), (11)^. Focusing on the situation in Japan, the national educational program for palliative care was started in 2008, and it mainly targets oncologists and palliative care physicians working at designated cancer hospitals. Although the Japanese opioid consumption in 2006 was very low and ranked at or near the bottom among a group of developed countries, it had achieved a less than 15% increase by 2010 ^(9)^. In this study, we first aimed to review the current situation regarding the adequacy of opioid consumption for cancer pain in each prefecture.

We also aimed to explore the determinants for enhancing the effect of education on opioid availability. Globally, adequate control of cancer pain and chronic non-cancer pain is increasingly viewed as a fundamental human right, and interest in effective pain management is diffusing outward into primary care ^(12), (13)^. Because of the long-term relationships that primary care physicians develop with their patients, they are generally tasked with managing the majority of patients with painful conditions ^(14)^. Further, shared coordinated care and transfer of care between oncologists at designated cancer hospitals and primary care physicians at local healthcare institutes have become common in Japan. We focused on the role of primary care physicians in managing cancer pain and prescribing opioids.

## Materials and Methods

Our local ethics committee (The Ethics Committee, Graduate School of Medicine, The University of Tokyo) approved this study [approval code: 3678-(2)]. The databases used in this study were open to public inspection, and all data were de-identified. The need for informed consent from each participant was therefore waived because of the anonymous nature of the data.

### Experiment 1

To address the adequacy of opioid availability for cancer pain in each prefecture in Japan, we calculated an index called the adequacy of consumption measure (AOM) ^(8)^. The AOM has been accepted and validated globally and is recommended to nation’s health authorities as the standard method of calculating the adequacy of opioid consumption by the WHO ^(15)^. The AOM indicates the morphine-equivalent dosage of opioid analgesics required for pain relief in terminal cancer patients in mg per capita based on the assumption that 80% of terminally ill cancer patients require a morphine-equivalent dose of 75 mg per person per day for an average of 90 days during the last year of their life ^(8)^.

We obtained the data from a public database released biennially by the Japanese Ministry of Health, Labour and Welfare (MHLW), in which the annual usage of morphine, oxycodone, and fentanyl in each prefecture is detailed ^(16)^. However, to the best of our knowledge, there are no formal data about opioid prescriptions for cancer pain specifically in Japan. The annual usage of opioids includes management for cancer pain and other indications. Morphine and fentanyl have several indications (for example, peri- and post-operative pain and chronic non-cancer pain) other than cancer pain in the Japanese insurance system. However, all forms of oxycodone have only been approved for cancer pain. In particular, the intravenous form of fentanyl is most frequently used for peri- and post-operative pain management, almost completely replacing morphine in this context. Intradermal fentanyl is used for both cancer pain and non-cancer chronic pain management. We presumed that the vast majority of fentanyl consumption is intravenous use for peri- and post-operative pain management. Morphine is certainly used for managing non-cancer pain in addition to cancer pain. However, we could speculate that such usage for non-cancer pain is relatively small (in other words, most morphine would be used for cancer pain management). Therefore, when calculating the AOM of cancer pain, we used morphine and oxycodone data, converted to a morphine-equivalent dose based on their equianalgesic dose ratios (i.e., oxycodone:morphine = 2:3) ^(4)^. The council for palliative care promotion, in which multisector actors and entities engage, convened by the Japanese MHLW, reported that morphine and oxycodone accounted for approximately 60% of morphine-equivalent opioid dosages for cancer pain management during the early 2010s as a result of their survey ^(17)^. We obtained annual cancer deaths in each prefecture from the annual public report by the National Cancer Center Japan. Combining these data together, we calculated the AOM in each prefecture in 2013 and 2015.

### Experiment 2

To explore the determinants for enhancing the effect of education on opioid availability, we obtained the annual prefectural healthcare expenditure from the nationwide database publicly released by the Japan Health Insurance Association, which is the largest health insurance society in Japan and covers 30% of the Japanese population ^(18)^. Among the items in the annual data, we focused on outpatient medical expenditures per day for hypertension and diabetes mellitus. These were the first (16%) and fourth (7%) most common disease entities in terms of the total number of outpatients and accounted for 83.6% of outpatients visiting primary care clinics in the early 2010s in Japan ^(19)^. As a matter of practical convenience, we considered the outpatient medical expenditure per day for hypertension and diabetes as a representative of primary care practice in local healthcare institutes. We contrasted the simple volume effects of these diseases to primary care practice. From the same database, we extracted items concerning the ratio of non-reassuring hypertension and diabetes to healthy individuals based on routine physical examination findings. Among local healthcare services, orthopedic practitioners are accustomed to managing chronic pain in general. We considered the outpatient medical expenditure for musculoskeletal disorders as representative practice for community pain services, which was the second most common disease entity in terms of total numbers of outpatients in the early 2010s in Japan ^(19)^. Further, we extracted data regarding outpatient medical expenditures per day for neoplasms from the database. Most of these were probably assumed to include expenses for diagnostic testing and anticancer treatment at outpatient clinics of designated cancer hospitals but not local healthcare institutes. As a matter of practical convenience, we considered the outpatient medical expenditure for neoplasms as representative practice of designated cancer hospitals. Using Spearman’s correlation analysis on the prefectural data from 2013 and 2015, we investigated the associations between the AOM and the three outpatient expenditures and local volumes of hypertension and diabetes.

## Results

### Experiment 1

**Figure 1.**
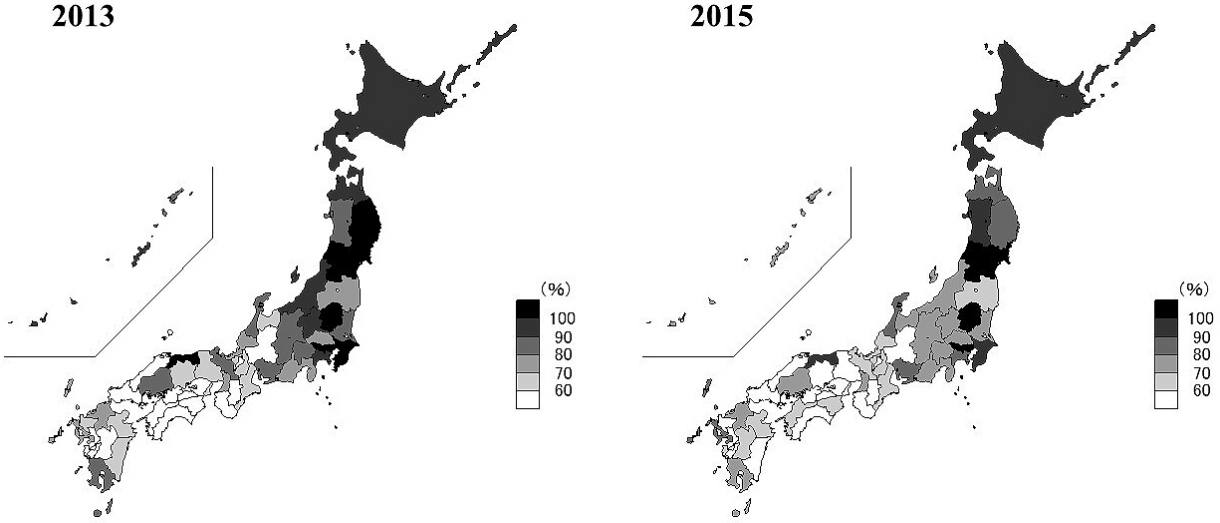
Percentage of the adequacy of opioid availability for cancer pain in each prefecture in 2013 and 2015. Bar scale depicts the adequacy of opioid availability for cancer pain based on the annual cancer deaths in each prefecture.

The Japanese nationwide results for the AOM were 78.2% ± 17.9% (mean ± standard deviation) and 73.8% ± 18.2% in 2013 and 2015, respectively (Figure 1). Only four prefectures (Yamagata, Tokyo, Tochigi, and Miyagi) achieved an AOM over 100% in both 2013 and 2015. The range of AOM between the best (Tottori, 117.3%) and worst (Wakayama, 50.3%) prefectures was 67.0% in 2013. In 2015, the gap was 88.6% between the best (Yamagata, 135.1%) and worst (Gifu, 46.6%) prefectures. Thus, the gap increased over two years. Changes in the AOM among prefectures varied widely: Yamagata demonstrated the best improvement of 32.0% (106.2% in 2013 to 135.1% in 2015), and Okinawa had the largest decrease of 23.9% (94.9% in 2013 to 71.5% in 2015).

### Experiment 2

**Figure 2.**
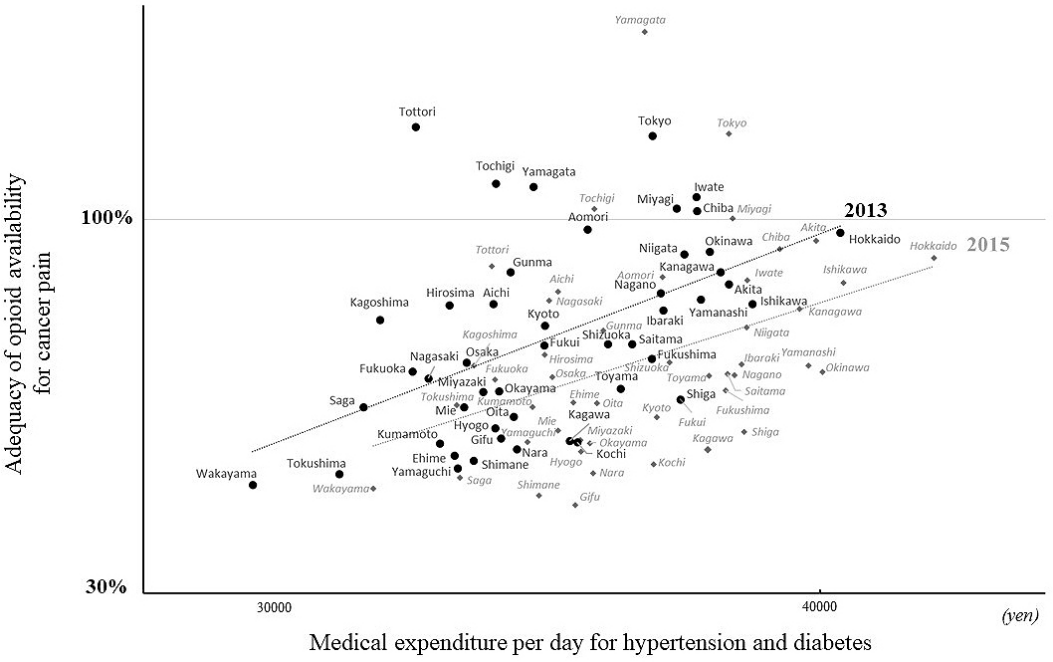
Scatterplots showing the relationship in individual prefectures between the adequacy of opioid availability for cancer pain and outpatient medical expenditure per day for hypertension and diabetes in 2013 and 2015. The adequacy indicates the opioids required for pain relief in terminal cancer patients in mg per capita. The medical expenditure represents primary care practice in local healthcare institutes. Black diamonds and gray circles represent the adequacy and expenditure of individual prefectures in 2013 and 2015, respectively. Spearman’s correlation analyses reveal the linear associations between them in 2013 (black line; p < 0.001, R = 0.50) and 2015 (gray dotted line; p = 0.006, R = 0.40).

In 2013, the AOM was significantly associated with outpatient medical expenditure per day for hypertension and diabetes (R = 0.50, p < 0.001; Figure 2), but not with the volume data (R = 0.12, p = 0.41). The linear correlation between the AOM and the medical expenditure was maintained in 2015 (R = 0.40, p = 0.006; Figure 2), but the association between the AOM and the volume effect did not reach significance (R = 0.31, p = 0.09). Therefore, the local risk holders of these diseases did not affect the pattern of opioid prescription for cancer pain, but primary care practice was firmly linked to it. The medical expenditure for musculoskeletal disorders was not associated with the AOM in either 2013 (R = 0.14, p = 0.34) or 2015 (R = −0.04, p = 0.79; Table 1). In addition, the medical expenditures for neoplasms was not associated with the AOM in either 2013 (R = 0.11, p = 0.44) or 2015 (R = 0.15, p = 0.32; Table 1).

**Table 1.** Correlations between the AOM of Cancer Pain and Outpatient Medical Expenditures.

	Medical expenditures per day for hypertension and diabetes		Medical expenditures per day for musculoskeletal disorders		Medical expenditures per day for neoplasms	
	R	*P value*	R	*P value*	R	*P value*
2013	0.50	*< 0.001*	0.14	*0.34*	0.11	*0.44*
2015	0.40	*0.006*	−0.04	*0.79*	0.15	*0.32*

P values are from the Spearman’s correlation analysis. R indicates correlation coefficients

## Discussion

The Japanese nationwide adequacy of opioid availability for terminally ill cancer patients was estimated to be 78.2% and 73.8% in 2013 and 2015, respectively. Differences in adequate opioid availability between prefectures were not lower than 65% in either year. To explore the determinants of such large gaps, we found that primary care practice, as typified by outpatient medical expenditure per day for hypertension and diabetes, is firmly associated with adequate opioid availability. These associations were independent of the volume of risk holders in the local community. Further, not only local orthopedic practice but also outpatient practice of designated cancer hospitals did not seem to be associated with adequate opioid availability. Our present findings indicate that primary care physicians are key regulators in cancer pain management.

For patients with cancer, especially those with advanced and incurable cancer, adequate pain relief is a central goal of care. To achieve this objective, healthcare professionals should have basic clinical competency for palliative care, assessing pain, and treating it with medications, including opioid analgesics, according to the individual needs of each patient ^(13)^. Opioid analgesics are critical to the effective relief of cancer pain. Effective treatment is predicated on sound assessments, individually tailored analgesic therapy, and the availability and accessibility of the required medications. Therefore, global initiatives (i.e., the WHO and International Narcotics Control Board) have continuously recommended and encouraged healthcare professionals, government, and health regulatory authorities to monitor and improve opioid consumption as a proxy of adequate opioid availability. Considering the recent global issues of a potential “opioid crisis,” especially in North America and Australia, the total opioid consumption should not necessarily be huge. However, in the present study, we calculated the adequacy of opioid consumption based on the number of cancer deaths. The adequacy of opioid consumption measure is based on terminally ill cancer patients not experiencing cancer-related pain and approaching death with dignity. Therefore, the adequacy of opioid consumption measure is recommended as the minimum goal to be achieved in all nations by the WHO ^(15)^. According to this WHO recommendation, opioid availability in Japan should doubtlessly increase as soon as possible. The council for palliative care promotion in Japan is reportedly recognized as one of the most active and advanced groups worldwide ^(20)^. However, opioid consumption seemed to plateau in Japan from the early 2000s to the early 2010s ^(21)^. Moreover, our findings demonstrate that the opioid consumption between 2013 and 2015 in Japan was approximately the same, if not slightly decreased, and did not follow the increase in cancer deaths. Further, apart from patients with cancer who are terminally ill, sufficient analgesic supplementation is still not provided to more than half of patients with cancer who have received and just completed anticancer treatment in Europe ^(22)^. Severe pain during cancer treatment could be an independent risk factor for worse cancer prognosis ^(23), (24)^. To the best of our knowledge, there are no data on how many cancer patients who have received and just completed anticancer treatment have cancer-treatment-related pain in Japan. By extrapolating the European situation to Japan, opioid availability in Japan may have to increase significantly to aggressively control both pain due to advanced cancer and cancer-treatment-related pain. Four prefectures achieved and surpassed the minimum goal of opioid availability for cancer pain management in both 2013 and 2015. Different from the globally established adequate opioid consumption measure for terminally ill cancer patients, the measure for patients who have received and just completed anticancer treatment has not yet been established worldwide. The United States and Canada, both of which have been in the midst of an opioid crisis this decade, had an adequacy of opioid consumption of 230% and 313% in 2010, respectively ^(9)^. Comparatively speaking, the achievement of opioid adequacy in four prefectures might not be an excess for managing not only cancer pain in terminally ill patients but also cancer-treatment-related pain in patients who are receiving or just completing anticancer treatment. Equally important, the rapid growth of opioid consumption for non-cancer pain should be carefully monitored. In Australia, which has also been in the midst of an opioid crisis in recent years, the opioid adequacy was 107% for cancer pain in 2010 ^(9)^. From the perspective of preventing the opioid crisis in Japan, Yamagata should be carefully monitored, in particular, because a rapid increase (32.0%) of opioid consumption between 2013 and 2015 was observed, and morphine, which has indications for not only cancer pain but also non-cancer pain, rather than oxycodone accounted for the majority of this increase.

By exploring determinants for enhancing opioid availability for cancer pain, we preliminarily identified primary care practice as its key regulator. In Japan, a vast majority of primary care physicians plausibly have knowledge of the prevailing principles of cancer pain treatment, the WHO three-step analgesic ladder, in which strong opioids are recommended as the most potent analgesics for moderate to severe pain ^(25)^. However, in clinical practice, it is sometimes difficult to titrate opioid analgesics to achieve pain relief possibly because of the inadequately low dosages of analgesics relative to the pain level ^(26)^. A systematic review of the barriers hindering adequate cancer pain management revealed not only inadequate assessment of pain but also healthcare professional’s inadequate knowledge of pain management ^(26)^. In particular, inadequate knowledge of opioid analgesics (e.g., effective dose, management of adverse effects, and likelihood of addiction or tolerance) is still described by many physicians and nurses. The latest US pain management guidelines ^(27)^, in which the necessities and risks of opioid medication for both cancer pain and chronic non-cancer pain management are expressed in a balanced manner, emphasize the critical role of primary care physicians because most patients can access primary care physicians more easily than oncologists, palliative care physicians, and pain specialists. Moreover, these guidelines emphasize that early consultation with either palliative care physicians or pain specialists and educational support, time, and financial resources for primary care physicians are essential for managing patients with painful conditions. On the basis of these recommendations and our present findings, the targets of the Japanese national educational program for palliative care should switch from oncologists working at designated cancer hospitals to primary care physicians. The context of the educational program should be more specialized to the practical use of opioid analgesics. Appropriate incentives might encourage primary care physicians to be more assertively involved in cancer pain management.

### Limitations

The present study was conducted based on databases open to public inspection. Opioid consumption data do not include information on diagnoses for opioid usage. As there are mostly specialized prescriptions for cancer pain, we extracted data on oxycodone and morphine prescriptions, fixed the share of morphine and oxycodone for cancer pain over prefectures, and calculated the adequacy of opioid availability using the council report. Thus, in Japan, there is no well-validated dataset demonstrating opioid consumption for specific clinical conditions. Such a precise dataset of opioid consumption for specific clinical conditions is required to further improve cancer care. Furthermore, the database of medical expenditure does not include the attributions of doctors and outpatient institutions (i.e., private clinics or outpatient clinics of local hospitals). We therefore indeterminately used the term “primary care physician” to generalize them. To more precisely specify the appropriate targets for the national Japanese educational program for palliative care, any future dataset of opioid consumption might need such attributions.

## Article Information

### Conflicts of Interest

None

### Sources of Funding

This work was supported by JSPS KAKENHI grant number [19H03749].

### Author Contributions

Kenji Azuma wrote the manuscript and collected and analyzed the whole data. Hiroaki Abe and Jun Hozumi assisted in writing the manuscript. Reo Inoue advised data analyses and interruptions from the standpoint of anesthesiology. Mitsuru Konishi advised data analyses and interruptions from the standpoint of gastroenterology. Rikuhei Tsuchida, Masae Ando, and Kosuke Saita assisted in data collection and data analyses. Masahiko Sumitani directed this study and reviewed the manuscript.

### Approval by Institutional Review Board (IRB)

The Ethics Committee, Graduate School of Medicine, The University of Tokyo, approval code 3678-(2)
